# A Fourth Order Entropy Stable Scheme for Hyperbolic Conservation Laws

**DOI:** 10.3390/e21050508

**Published:** 2019-05-19

**Authors:** Xiaohan Cheng

**Affiliations:** School of Science, Chang’an University, Xi’an 710064, China; xhcheng@chd.edu.cn

**Keywords:** conservation laws, entropy stable, entropy conservative, non-oscillatory reconstruction, sign property

## Abstract

This paper develops a fourth order entropy stable scheme to approximate the entropy solution of one-dimensional hyperbolic conservation laws. The scheme is constructed by employing a high order entropy conservative flux of order four in conjunction with a suitable numerical diffusion operator that based on a fourth order non-oscillatory reconstruction which satisfies the sign property. The constructed scheme possesses two features: (1) it achieves fourth order accuracy in the smooth area while keeping high resolution with sharp discontinuity transitions in the nonsmooth area; (2) it is entropy stable. Some typical numerical experiments are performed to illustrate the capability of the new entropy stable scheme.

## 1. Introduction

During the past few decades, abundant numerical methods for solving hyperbolic conservation laws have been designed; one can consult the review papers [1,2] and the references therein. Among the various methods, high order schemes, such as total variation diminishing (TVD) schemes, weighted essentially non-oscillatory (WENO) schemes and discontinuous Galerkin (DG) schemes, have achieved great success. However, it seems that there are few entropy stability results for high order numerical schemes for hyperbolic conservation laws, especially for the nonlinear hyperbolic conservation laws. In view of the above discussion, we limit our research on high order entropy stable methods for solving hyperbolic conservation laws.

In accordance with the idea of Tadmor, entropy schemes are often constructed by utilizing entropy conservative flux in conjunction with suitable numerical diffusion [3]. For example, physical viscosity was discretized and used as numerical diffusion in [4]. Roe-type numerical diffusion was selected for various systems like Euler equations, shallow water equations and ideal magnetohydrodynamic equations [5,6,7]. Based on the limiter mechanism, Liu et al. constructed a family of entropy consistent schemes with high resolution [8]. Recently, Dubey discussed the amount of suitable diffusion for the sake of devising non-oscillatory entropy stable schemes in the TVD sense [9]. To obtain high order entropy stable schemes, Fjordholm et al. firstly proved that ENO reconstruction satisfies a sign property and presented entropy stable schemes based on ENO reconstruction of arbitrary order accuracy [10,11]. To overcome the drawbacks of ENO reconstruction, we presented a third order reconstruction which is non-oscillatory and satisfies the sign property to construct a third order entropy stable scheme [12]. WENO reconstruction was modified to satisfy the sign property and also used to construct an entropy stable scheme in [13,14], although they are limited to third order accuracy.

In this paper, we are aiming at presenting a new entropy stable scheme of fourth order accuracy to solve hyperbolic conservation laws in one dimension. First, we employ a fourth order entropy conservative flux which is a linear combination of two-point entropy conservative fluxes. Then, we present a non-oscillatory reconstruction of fourth order accuracy which possesses the sign property to obtain a fourth order accurate numerical diffusion operator. By adding this numerical diffusion operator to the entropy conservative flux, the resulting flux is entropy stable.

The remainder of this paper is given as follows. Section 2 describes the procedure for building our fourth order entropy scheme. After that, we present some typical numerical experiments to show the effectiveness of the newly developed scheme. Finally, concluding remarks are given in Section 4.

## 2. Numerical Method

Consider the scalar conservation law

(1) $$\frac{\partial }{\partial t}u+\frac{\partial }{\partial x}f\left(u\right)=0.$$

The weak solutions for Equation (1) are not unique and we are interested in the so-called entropy solution, which satisfies the entropy condition

(2) $$\frac{\partial }{\partial t}\eta \left(u\right)+\frac{\partial }{\partial x}q\left(u\right)\leq 0.$$

Here, the entropy function η(u) is convex and the entropy flux function q(u) satisfies $\frac{\partial q}{\partial u}=v\frac{\partial f}{\partial u}$ with the entropy variable $v=\frac{\partial \eta }{\partial u}$. To solve Equation (1) with the conservative difference method, we have the semi-discrete scheme

(3) $$\frac{du_{i}}{dt}=-\frac{f_{i+1/2}-f_{i-1/2}}{\Delta x},$$

where $u_{i}$ represents the point valve at the node $x_{i}$ on a uniform Cartesian mesh with the mesh size $x_{i+1}-x_{i}=\Delta x$. The scheme (3) is said to be entropy stable if it satisfies a discrete version of the entropy condition (2), namely,

(4) $$\frac{\partial }{\partial t}\eta \left(u_{i}\right)+\frac{q_{i+1/2}-q_{i-1/2}}{\Delta x}\leq 0;$$

the scheme is said to be entropy conservative if it satisfies the discrete entropy equality

(5) $$\frac{\partial }{\partial t}\eta \left(u_{i}\right)+\frac{q_{i+1/2}-q_{i-1/2}}{\Delta x}=0.$$

In this work, we utilize the familiar Runge–Kutta scheme of order four to solve the ordinary Equation (3). Now, we focus on how to build the entropy stable numerical flux $f_{i+1/2}$.

First, the numerical flux is split into an entropy conservative part and a numerical diffusion part

(6) $$f_{i+1/2}=f_{i+1/2}^{EC}-\frac{1}{2}\delta_{i+1/2}\left(v_{i+1/2}^{+}-v_{i+1/2}^{-}\right).$$

Here, $f_{i+1/2}^{EC}$ is a fourth order entropy conservative flux [15] expressed as

(7) $$f_{i+1/2}^{EC}=\frac{4}{3}\tilde{f}\left(u_{i},u_{i+1}\right)-\frac{1}{6}\left(\tilde{f}\left(u_{i-1},u_{i+1}\right)-\tilde{f}\left(u_{i},u_{i+2}\right)\right)$$

with $\tilde{f}\left(\alpha ,\beta \right)$ being the second order entropy conservative flux defined by Tadmor [3]. Generally, $\delta_{i+1/2}$ in the numerical diffusion part is chosen to be $\left|f^{\prime }\left(u_{i+1/2}\right)\right|$. If the reconstructed values $v_{i+1/2}^{\pm }$ at the interfaces from the entropy variable $v_{i}$ satisfy

(8) $$sign\left(v_{i+1/2}^{+}-v_{i+1/2}^{-}\right)=sign\left(v_{i+1}-v_{i}\right),$$

the numerical flux (6) achieves entropy stability. Property (8) in which the jump of the reconstructed point values at each cell interface has the same sign as the jump of the underlying point values across that interface is called the sign property. We present the fourth order reconstruction satisfying the sign property as follows to obtain $v_{i+1/2}^{\pm }$.

Consider a polynomial reconstruction of the form

(9) $$p_{i}\left(x\right)=v_{i}+d_{i}\left(\frac{x-x_{i}}{\Delta x}\right)+\left(\frac{v_{i-1}-2v_{i}+v_{i+1}}{2}\right){\left(\frac{x-x_{i}}{\Delta x}\right)}^{2}+\left(\frac{-v_{i-1}+v_{i+1}-2d_{i}}{2}\right){\left(\frac{x-x_{i}}{\Delta x}\right)}^{3}$$

with $d_{i}$ being a slope function. For convenience, we introduce the following notation

(10) $$\begin{array}{c} ds_{i}=\frac{2}{3}v_{i+1}-\frac{2}{3}v_{i-1}-\frac{1}{12}v_{i+2}+\frac{1}{12}v_{i-2},\\ WC_{i}=v_{i+1}-v_{i-1},\;WR_{i}=v_{i+1}-v_{i},\;WC2_{i}=v_{i+2}-v_{i-2},\\ ds1_{i}=0,\;ds2_{i}=\frac{1}{2}\left(WC_{i}-8WR_{i}\right),\;ds3_{i}=\frac{1}{2}\left(8WR_{i}-7WC_{i}\right),\\ S_{i}=sign\left(WC_{i}\right),\\ C_{1}=\frac{\sqrt{3}}{6},\;C_{2}=\frac{6}{12+\sqrt{3}},\; \end{array}$$

and define the slope $d_{i}$ in the following way:

1. If $S_{i}=0$, then $d_{i}=0$.
2. If $S_{i}\neq 0$ and $\left(2S_{i}\cdot WC_{i}\geq S_{i}\cdot WC2_{i}\right)$, then $d_{i}=ds_{i}$.
3. If $S_{i}\neq 0$ and $\left(2S_{i}\cdot WC_{i}<S_{i}\cdot WC2_{i}\right)$, then the following hold:
  (a) If $v_{i}=\frac{v_{i+1}+v_{i-1}}{2}$, we define
    $$d_{i}=\left\{\begin{array}{cc} \max(ds1_{i},ds_{i}), & \mathrm{if}\;S_{i}>0,\\ \min(ds1_{i},ds_{i}), & \mathrm{if}\;S_{i}<0. \end{array}\right.$$
  (b) If $v_{i}\neq \frac{v_{i+1}+v_{i-1}}{2}$, then the following hold:
    i. If $\left|WR_{i}-\frac{1}{2}WC_{i}\right|\geq \frac{1}{8}\left|WC2_{i}-2WC_{i}\right|$, then
      $$d_{i}=\left\{\begin{array}{cc} \max(ds2_{i},ds3,ds_{i}), & \mathrm{if}\;S_{i}>0,\\ \min(ds2_{i},ds3,ds_{i}), & \mathrm{if}\;S_{i}<0. \end{array}\right.$$
    ii. If $\left|WR_{i}-\frac{1}{2}WC_{i}\right|<\frac{1}{8}\left|WC2_{i}-2WC_{i}\right|$, then
      $$d_{i}=\left\{\begin{array}{cc} \frac{WC_{i}}{2}-S_{i}\cdot C1\cdot \left|2WR_{i}-WC_{i}\right|, & \;\mathrm{if}\;\left|\frac{WR_{i}}{WC_{i}}-\frac{1}{2}\right|\leq C2,\\ \frac{WC_{i}}{2}, & \;\mathrm{if}\;\left|\frac{WR_{i}}{WC_{i}}-\frac{1}{2}\right|>C2. \end{array}\right.$$

**Theorem** **1.** *With this definition of the slope, the polynomial reconstruction* (9) *fulfills the shape preserving property, namely,*

- $p_{i}\left(x\right)$ is monotone in $\left[x_{i-1/2},x_{i+1/2}\right]$ iff the point values $\left\{v_{i-1},v_{i},v_{i+1}\right\}$ are;
- $p_{i}\left(x\right)$ generates an extremum in the interior of $\left[x_{i-1/2},x_{i+1/2}\right]$ iff $v_{i}$ is an extremum value.

The proof of this theorem can be found in [16]. According to this property, the polynomial $p_{i}\left(x\right)$ does not create new extrema in the interior of $\left[x_{i-1/2},x_{i+1/2}\right]$. In other words, the polynomial $p_{i}\left(x\right)$ may create new extrema at the interfaces $\left\{x_{i\pm 1/2}\right\}$. To keep away from such spurious extrema, we can employ a similar strategy as in [17]. Let us modify the polynomial reconstruction to be the following form:

(11) $$\varphi_{i}\left(x\right)=\left(1-\theta_{i}\right)v_{i}+\theta_{i}p_{i}\left(x\right),\;0<\theta_{i}<1.$$

The limiter $\theta_{i}$ is determined by

(12) $$\theta_{i}=\left\{\begin{array}{cc} \min\left\{\frac{M_{i+1/2}-v_{i}}{M_{i}-v_{i}},\frac{m_{i-1/2}-v_{i}}{m_{i}-v_{i}},1\right\}, & \mathrm{if}\;v_{i-1}<v_{i}<v_{i+1},\\ \min\left\{\frac{M_{i-1/2}-v_{i}}{M_{i}-v_{i}},\frac{m_{i+1/2}-v_{i}}{m_{i}-v_{i}},1\right\}, & \mathrm{if}\;v_{i-1}>v_{i}>v_{i+1},\\ 1, & \mathrm{otherwise}, \end{array}\right.$$

with

$$M_{i}=\max_{x\in [x_{i-1/2},x_{i+1/2}]}p_{i}\left(x\right),\;m_{i}=\min_{x\in [x_{i-1/2},x_{i+1/2}]}p_{i}\left(x\right),$$

and

$$\left\{\begin{array}{c} M_{i\pm 1/2}=\max\left\{\frac{1}{2}\left(v_{i}+v_{i\pm 1}\right),p_{i\pm 1}\left(x_{i\pm 1/2}\right)\right\},\\ m_{i\pm 1/2}=\min\left\{\frac{1}{2}\left(v_{i}+v_{i\pm 1}\right),p_{i\pm 1}\left(x_{i\pm 1/2}\right)\right\}. \end{array}\right.$$

**Theorem** **2.** *The polynomial reconstruction* (11) *is fourth order accurate and fulfills the sign property* (8).

**Theorem** **3.** *With the definitions of the reconstructed values*$v_{i+1/2}^{+}=\varphi_{i+1}\left(x_{i+1/2}\right)$*and*$v_{i+1/2}^{-}=\varphi_{i}\left(x_{i+1/2}\right)$, *the numerical flux* (6) *is entropy stable and fourth order accurate.*

**Remark** **1.** *The proofs for Theorems 2 and 3 can be carried out very similarly as in [12,17]. We omit the simple but trivial procedure here.*

**Remark** **2.** *For hyperbolic systems, the reconstruction procedure should be implemented in the local characteristic directions for the purpose of achieving entropy stability. The detailed steps can be found in our previous paper [12].*

## 3. Numerical Examples

In this section, we illustrate the effectiveness of the presented scheme which is abbreviated by ES4 by means of three typical examples. Numerical results include the convergence order and the capacity of dealing with discontinuous problems.

**Example** **1.** *Consider the linear advection equation*$u_{t}+u_{x}=0$*on the domain*[-1,1]*with two initial data*u(x,0)=sin(πx)*and*$u\left(x,0\right)=sin^{4}\left(\pi x\right)$. *For a comparison, a third order entropy stable scheme (ES3) [12] is also implemented for this example. The numerical errors (*$L^{1}$*error and*$L^{\infty }$*error) and convergence orders are displayed in Table 1 and Table 2. We can observe that the fourth order convergence of the proposed scheme is confirmed and ES4 performs better than ES3.*

**Example** **2.** *Consider the Burgers equation*

(13) $$u_{t}+{\left(u^{2}/2\right)}_{x}=0$$

*subjected to the initial data*

$$u\left(x,0\right)=\left\{\begin{array}{cc} 1, & \mathrm{for}\;|x|\leq 1/3,\\ -1, & \mathrm{for}\;1/3<|x|\leq 1. \end{array}\right.$$

**Figure 1:**
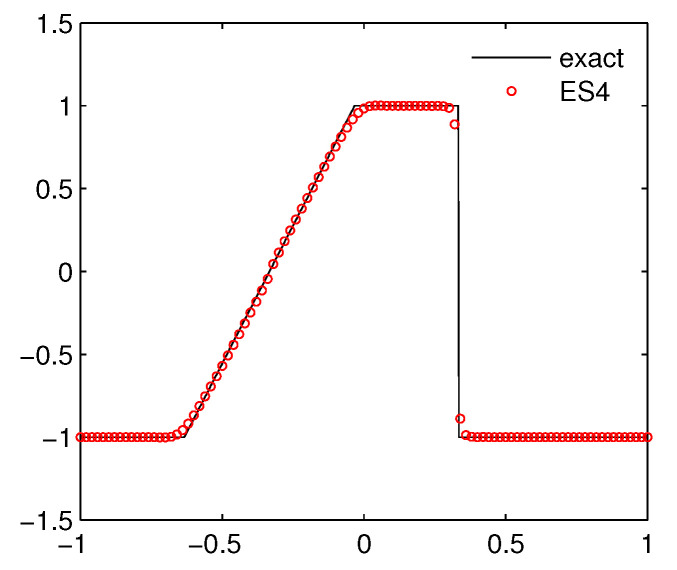
The numerical result for Burgers equation.

*For this problem, we can deduce the analytical solution that evolves a rarefaction fan and a stationary shock on the left-hand and right-hand side, respectively. Figure 1 presents the numerical result at time*t=0.3*on a mesh of* 100 *grids. Our scheme resolves the shock wave and the rarefaction wave very well.*

**Example** **3.** *Consider the Euler equations from aerodynamics*

(14) $$\frac{\partial }{\partial t}\left(\begin{array}{c} \rho \\ \rho \mu \\ E \end{array}\right)+\frac{\partial }{\partial x}\left(\begin{array}{c} \rho \mu \\ \rho \mu^{2}+p\\ \mu (E+p) \end{array}\right)=0$$

*with ρ,μ,p and E being the density, velocity, pressure and total energy, respectively. For an idea gas, the total energy E is given by the relation*

(15) $$E=\frac{p}{\gamma -1}+\frac{1}{2}\rho \mu^{2}$$

*with the specific heats ratio*γ=1.4. *Three Riemann problems are tested by the presented scheme.*

Case 1: Sod’s shock tube problem. The initial data is given as

$$\left(\rho ,\mu ,p\right)=\left\{\begin{array}{cc} (1,0,1), & \mathrm{for}\;x<0,\\ (0.125,0,0.1), & \mathrm{for}\;x>0. \end{array}\right.$$

**Figure 2:**
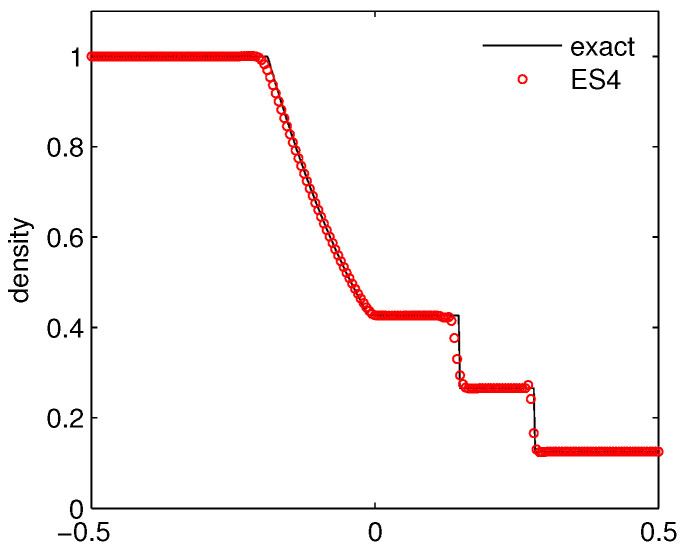
The density for Sod’s shock tube problem.

The numerical simulation is carried out on a mesh of 200 grids on [-0.5,0.5] up to time t=0.16. The computed density is plotted in Figure 2. We can see that the ES4 scheme performs well by capturing the shock, the contact discontinuity and the rarefaction wave accurately.

Case 2: Toro’s 123 problem. The initial data is given as

$$\left(\rho ,\mu ,p\right)=\left\{\begin{array}{cc} (1,-2,0.4), & \mathrm{for}\;x<0,\\ (1,2,0.4), & \mathrm{for}\;x>0. \end{array}\right.$$

**Figure 3:**
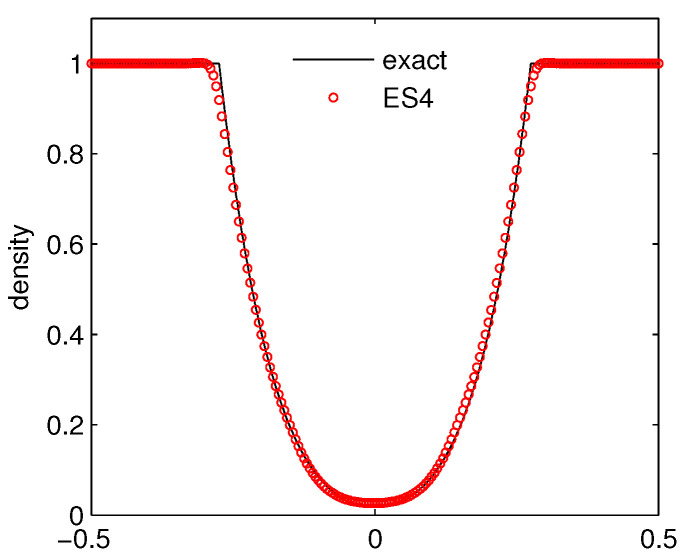
The density for Toro’s 123 problem.

The difficulty for simulating this problem lies in the fact that the pressure between the evolved rarefactions is very small (near vacuum) and may bring about the blow-ups of the code if the numerical method is not robust. The numerical simulation is carried out on a mesh of 200 grids on [-0.5,0.5] up to time t=0.1. Figure 3 displays the computed density. It can be observed that the computed result by ES4 compares well with the reference solution.

Case 3: Shu–Osher problem. The initial data is given as

$$\left(\rho ,\mu ,p\right)=\left\{\begin{array}{cc} (3.857,2.629,10.333), & \mathrm{for}\;x<-4,\\ (1+0.2\sin(5x),0,1), & \mathrm{for}\;x>-4. \end{array}\right.$$

**Figure 4:**
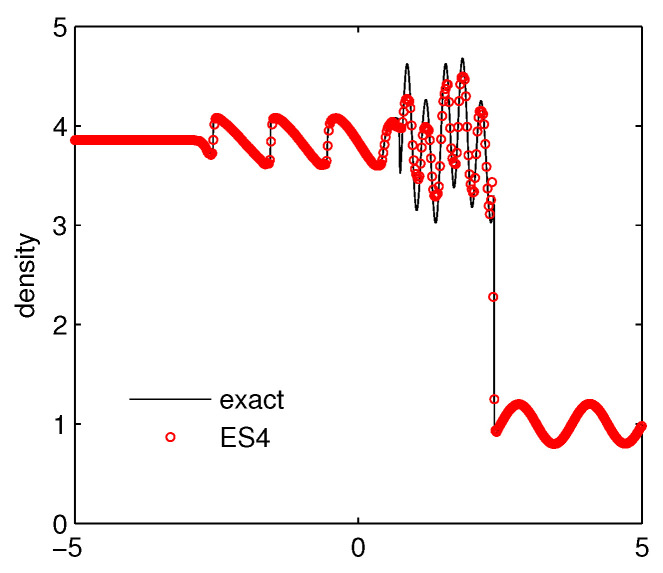
The density for the Shu–Osher problem.

This problem, also called the shock density-wave interaction problem, describes a moving Mach 3 shock interacting with sine waves in density. The numerical simulation is carried out on a mesh of 500 grids on [-5,5] up to time t=1.8. We present the results of density in Figure 4. It can be seen clearly that the ES4 scheme produces accurate results and captures the sine wave well.

## 4. Conclusions

This paper presents a fourth order entropy stable scheme for solving one-dimensional hyperbolic conservation laws. Along the lines of [10,12], our scheme is also obtained by utilizing entropy conservative flux in conjunction with suitable numerical diffusion. We first select the existing fourth order entropy conservative scheme based on the combination of the two-point entropy conservative flux. The main novelty lies in the construction of numerical diffusion by presenting a fourth order non-oscillatory reconstruction possessing the sign property. Compared to other high order schemes, the main advantage of our scheme is the entropy stability. Some numerical results are displayed to show the accuracy and shock capturing capacity of our scheme. Ongoing work involves generalizing the idea of this paper to multidimensional cases and other hyperbolic systems such as shallow water equations and magnetohydrodynamic equations.

## Figures and Tables

**Table 1:** The numerical errors and convergence orders for $u_{t}+u_{x}=0$ with u(x,0)=sin(πx) at t=8.

Method	*N*	$L^{1}$ Error	$L^{1}$ Order	$L^{\infty }$ Error	$L^{\infty }$ Order
	40	7.8941 $\times 10^{-3}$	2.9838	6.1563 $\times 10^{-3}$	2.9528
	80	9.8686 $\times 10^{-4}$	2.9999	7.7371 $\times 10^{-4}$	2.9922
ES3	160	1.2332 $\times 10^{-4}$	3.0004	9.6812 $\times 10^{-5}$	2.9985
	320	1.5413 $\times 10^{-5}$	3.0002	1.2104 $\times 10^{-5}$	2.9997
	640	1.9266 $\times 10^{-6}$	3.0000	1.5131 $\times 10^{-6}$	2.9999
	40	7.2962 $\times 10^{-4}$		5.5350 $\times 10^{-4}$	
	80	4.4746 $\times 10^{-5}$	4.0273	3.4513 $\times 10^{-5}$	4.0034
ES4	160	2.7650 $\times 10^{-6}$	4.0164	2.1523 $\times 10^{-6}$	4.0032
	320	1.7178 $\times 10^{-7}$	4.0086	1.3430 $\times 10^{-7}$	4.0023
	640	1.0702 $\times 10^{-8}$	4.0046	8.3862 $\times 10^{-9}$	4.0013

**Table 2:** The numerical errors and convergence orders for $u_{t}+u_{x}=0$ with $u\left(x,0\right)=sin^{4}\left(\pi x\right)$ at t=1.

Method	*N*	$L^{1}$ Error	$L^{1}$ Order	$L^{\infty }$ Error	$L^{\infty }$ Order
	80	3.8419 $\times 10^{-3}$		3.7835 $\times 10^{-3}$	
	160	4.9986 $\times 10^{-4}$	2.9422	5.8676 $\times 10^{-4}$	2.6889
ES3	320	6.7841 $\times 10^{-5}$	2.8813	8.6901 $\times 10^{-5}$	2.7553
	640	1.0357 $\times 10^{-5}$	2.7115	1.5669 $\times 10^{-5}$	2.4715
	1280	1.4533 $\times 10^{-6}$	2.5625	4.9399 $\times 10^{-6}$	1.6654
	80	8.1572 $\times 10^{-4}$		1.2001 $\times 10^{-3}$	
	160	5.9041 $\times 10^{-5}$	3.7883	1.4743 $\times 10^{-4}$	3.0249
ES4	320	4.9006 $\times 10^{-6}$	3.5907	2.7023 $\times 10^{-5}$	1.3202
	640	1.6184 $\times 10^{-7}$	4.9203	1.4716 $\times 10^{-7}$	7.5207
	1280	1.0013 $\times 10^{-8}$	4.0146	8.6289 $\times 10^{-9}$	4.0921
